# Diverse pro-inflammatory endotoxin recognition systems of mammalian innate immunity

**DOI:** 10.12688/f1000research.13977.1

**Published:** 2018-04-30

**Authors:** Jerrold Weiss, Jason Barker

**Affiliations:** 1 Inflammation Program and Departments of Internal Medicine and Microbiology, University of Iowa, Iowa City, Iowa, USA; 2 Veterans Affairs Medical Center, Iowa City, Iowa, USA

**Keywords:** lipopolysaccharide, TLR4, MD-2, CD14, LBP, caspase-4, caspase-5, caspase-11, non-canonical inflammasome

## Abstract

In humans and other mammals, recognition of endotoxins—abundant surface lipopolysaccharides (LPS) of Gram-negative bacteria—provides a potent stimulus for induction of inflammation and mobilization of host defenses. The structurally unique lipid A region of LPS is the principal determinant of this pro-inflammatory activity. This region of LPS is normally buried within the bacterial outer membrane and aggregates of purified LPS, making even more remarkable its picomolar potency and the ability of discrete variations in lipid A structure to markedly alter the pro-inflammatory activity of LPS. Two recognition systems—MD-2/TLR4 and “LPS-sensing” cytosolic caspases—together confer LPS responsiveness at the host cell surface, within endosomes, and at sites physically accessible to the cytosol. Understanding how the lipid A of LPS is delivered and recognized at these diverse sites is crucial to understanding how the magnitude and character of the inflammatory responses are regulated.

## Introduction

Endotoxins are abundant surface lipopolysaccharides (LPS) of Gram-negative bacteria (GNB) that can potently induce both protective and potentially harmful inflammatory responses in humans and other mammals ^1^. Two different recognition systems—MD-2/TLR4 and “LPS-sensing” cytosolic caspases—together confer responsiveness to LPS at the host cell surface, within endosomes, and in the cytosol ^2, 3^. The structurally unique lipid A region of LPS that is normally embedded within the GNB outer membrane (OM) is the principal determinant of both MD-2/TLR4 and caspase activation ^1, 4– 6^. Detailed studies of the MD-2/TLR4 system have revealed the importance of other host proteins in extraction and delivery of activating LPS monomers to MD-2/TLR4 ^7– 9^. Whether or not analogous alterations of LPS are needed in its presentation to LPS-sensing caspases is unknown. With few exceptions, the molecular and structural details that determine the range and limitation of pattern recognition of LPS are also unknown. These structure–function relationships have important implications both for a better understanding of the physiological and pathophysiologic roles of these systems and for ongoing efforts to develop novel immune modulators.

The foci of this brief review are evolving knowledge, concepts, and questions concerning the molecular and structural determinants of delivery of activating LPS and the requirements for engagement and activation by LPS of MD-2/TLR4 and inflammatory caspases. Several recent reviews provide excellent and complementary discussion of the regulation of downstream signal transduction and cellular responses triggered by LPS ^10– 14^.

## Modifications of LPS that promote recognition by and activation of MD-2/TLR4

**Figure 1.**
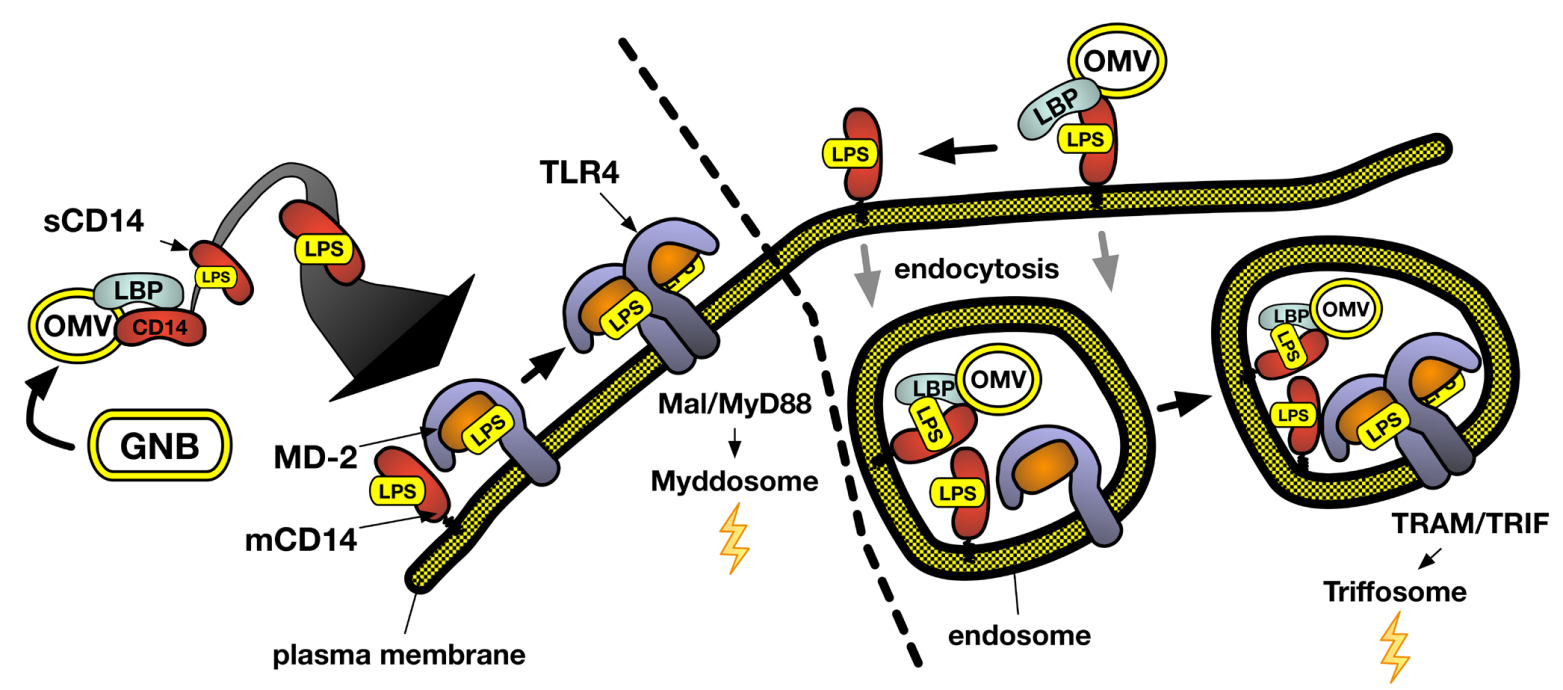
Molecular and subcellular requirements for activation of MyD88-dependent or TRIF-dependent signaling by picomolar LPS. Concerted LBP/CD14 action can convert one LPS-rich particle (for example, OMV) into thousands of TLR4-activating monomeric LPS.CD14 complexes, amplifying the potency of LPS toward MD-2/TLR4. Variables in lipid A/LPS structure could affect LPS potency by affecting the efficiency of extraction, delivery, and binding of activating LPS monomers to MD-2/TLR4 or the efficiency of LPS.MD-2.TLR4 dimerization or both. Variables affecting the relative rates of LPS.MD-2.TLR4 dimerization, assembly (or turnover) of Mal/MyD88, and/or endocytosis of the ternary complex or LPS-rich particles (for example, OMVs) may regulate the induction of MyD88-dependent signaling versus TRIF-dependent signaling. GNB, Gram-negative bacteria; LBP, lipopolysaccharide-binding protein; LPS, lipopolysaccharide; OMV, outer membrane vesicle; TRAM, TRIF-related adaptor molecule; TRIF, TIR domain-containing adapter-inducing interferon-β.

Activation of MD-2/TLR4 by LPS requires binding of an individual LPS molecule (LPS monomer) to MD-2/TLR4 ^15^ and dimerization of the LPS.MD-2.TLR4 ternary complex ^16^. Alone, MD-2/TLR4 has limited affinity for LPS-rich interfaces in which LPS naturally resides and no ability to extract LPS monomers from these surfaces ^17^. Hence, ancillary proteins are required to extract and deliver LPS monomers from the GNB OM or from aggregates of purified LPS (LPS-agg) to MD-2/TLR4 ^18^ ( Figure 1). The concerted action of extracellular LPS-binding protein (LBP) with soluble (s) or glycosylphosphatidylinositol (GPI)-linked membrane (m)CD14 provides the most efficient mechanism but not an exclusive one ^7^. When LPS is a potent MD-2/TLR4 agonist (for example, lipid A region contains hexa-acylated bis-phosphorylated diglucosamine), as few as 25 LPS.MD-2.TLR4 complexes per cell can trigger measurable pro-inflammatory responses, implying very efficient dimerization of these ternary complexes ^19^. Less potent ternary complexes containing discrete structural variants of LPS or MD-2 (or both) require higher surface concentrations to induce TLR4-dependent cell activation, implying less-efficient ternary complex dimerization ^20^.

New insights gained from Ryu *et al*. ^9^ confirmed the required order of reactions of LPS with LBP, CD14, and MD-2/TLR4 ^8, 17^ and revealed how LBP, in concert with CD14, catalyzes the release of LPS monomers from an LPS-rich interface. LBP, at concentrations substoichiometric to LPS, induces the rearrangement of LPS within the interface in the absence of CD14 that may be necessary but not sufficient for release of LPS monomers ^21, 22^. Release of LPS monomers requires the simultaneous presence of bound CD14, so that extraction of an LPS monomer from the interface is concomitant with transfer to CD14 ^9^. As predicted from earlier biochemical and structural studies ^21, 23^, LBP binds to the LPS-rich surface via the extended end of its N-terminal domain and to CD14 via its C-terminal domain at the opposite end of LBP ^9^. The extended geometry of LBP (>100 Å) ^24^ may be important when LPS and CD14 are part of separate (bacterial and host) surfaces.

This ordered pathway reflects the distinct “LPS recognition” properties of LBP, CD14, MD-2, and TLR4 that depend on the ability of the same LPS molecule to exist in different physical states via distinct intermolecular associations. Of these four proteins, only LBP has high (picomolar to nanomolar) affinity for LPS-rich interfaces ^25, 26^, in which LPS molecules at a high density are packed in close proximity. This permits LBP to act at tissue sites soon after GNB invasion when and where both LBP and LPS (GNB) concentrations are low. The increased avidity of CD14 for an LBP-modified LPS interface likely reflects combined CD14–LPS and CD14–LBP interactions ^9^. Partial insertion of fatty acyl chains of an individual LPS molecule into a hydrophobic pocket of CD14 ^22, 27^ is followed by dis-engagement of CD14 from the LPS interface and dissociation from LBP, permitting repeated cycles of additional CD14 binding and formation and release of monomeric LPS.CD14 complexes ^9^. Elevated LBP concentrations during the acute-phase response inhibit formation and release of monomeric LPS.CD14 ^22^ and transfer of LPS from CD14 to MD-2/TLR4 ^28^. Whereas early studies of MD-2 interactions with purified LPS (that is, LPS-agg) reported a K _D_ of about 65 nM ^29^, LPS transfer from monomeric LPS.CD14 to either MD-2 alone or MD-2/TLR4 has an apparent K _D_ of about 500 pM ^7, 15^. MD-2 must be monomeric to interact with LPS.CD14 ^30^, explaining the apparent “TLR4 dependence” of LPS binding to MD-2 when MD-2 is produced under conditions unfavorable for its secretion or stability as an isolated monomer ^9, 31, 32^. The transfer of LPS from a hydrophobic pocket of CD14 to that of MD-2(/TLR4) at picomolar concentrations is presumably facilitated by transient CD14–MD-2 interactions. Subsequent receptor activation does not require retention of CD14 as part of an activated receptor complex with MD-2/TLR4 ^15, 17^. Formation of a monomeric LPS.MD-2.TLR4 ternary complex is driven by TLR4-independent LPS–MD-2 interactions ^17, 33, 34^ and LPS-independent MD-2–TLR4 interactions ^16, 19^, each with picomolar affinity. In contrast, LPS.MD-2.TLR4 dimerization is driven by agonist-dependent LPS–TLR4 and MD-2–TLR4 contacts between two ternary complexes ^16^, dependent on both the structure and the configuration of LPS (lipid A) bound to MD-2 and of MD-2 itself at the dimerization interface ^16, 35– 37^. In sum, while LBP, CD14, MD-2, and TLR4 are each “lipid A (LPS) recognition” proteins, the structural bases of molecular recognition of LPS by each protein are different, depending on both the physical presentation of LPS (for example, interface, modified interface, and monomer) and the host protein with which LPS is presented.

## “Pattern recognition” by MD-2/TLR4

Different GNB can produce structurally different LPS, and an individual GNB can produce different LPS under different environmental circumstances ^38– 40^. From the perspective of both innate immunity and bacterial pathogenesis, the range and limits of pattern recognition by MD-2/TLR4 (and those host proteins regulating LPS presentation to MD-2/TLR4) are of crucial importance ^3, 41^. Variations in lipid A acylation (number, length, saturation, and geometry) and charged and uncharged polar substituents within and neighboring the lipid A region each have been shown to affect MD-2/TLR4 agonist (or antagonist) potency ^42– 45^. However, it has been difficult to establish precise structure–activity relationships (SARs), in part because of the structural heterogeneity of virtually all “purified” LPS derived from bacteria. To a limited extent, this analytical challenge has been circumvented by the development of novel bacterial libraries in which defined bacterial lipid A-modifying enzymes, alone or in tandem, have been cloned into a parent strain rendered devoid of these enzymes ^42, 44^. In parallel, progress in the synthesis of lipid A mimetics has offered the promise of more extensive and precise SAR studies, albeit with the introduction of structural features not present in bacterial lipid A/LPS ^46^. To date, efforts to apply insights from structural biology and molecular modeling analyses of ligand.MD-2 and ligand.MD-2.TLR4 complexes to SAR ^47, 48^ have been hampered by the use of functional assays that cannot discriminate between effects on extraction, delivery, and binding of ligand monomers to MD-2/TLR4 versus the agonist potency per se of the ligand.MD-2.TLR4 ternary complex. Thus far, the agonist potencies of only tetra-, penta-, and hexa-acylated lipid A/LPS.MD-2.TLR4 ternary complexes have been directly compared ^49^. This comparison demonstrated a pronounced (>>100-fold) and selective effect of LPS acylation on ternary complex agonist potency (hexa >> tetra) correlating with acylation-dependent effects on the configuration of bound LPS and MD-2 ^16, 37^ and the agonist versus antagonist properties of hexa-acylated versus tetra-acylated lipid A/LPS toward human MD-2/TLR4.

## Regulation of induction of MyD88-dependent and TRIF-dependent signal transduction

Signaling from activated MD-2/TLR4 can occur both at the cell surface and within endosomes ^10, 12^ ( Figure 1). Signaling initiated at the cell surface is linked to assembly of TIRAP (Mal) and MyD88 to the cytoplasmic region of TLR4 following dimerization of LPS.MD-2.TLR4 at the cell surface. In contrast, signaling dependent on assembly of TRIF (TIR domain-containing adapter-inducing interferon-β) and TRAM (TRIF-related adaptor molecule) occurs exclusively at endosomes that contain activated MD-2/TLR4 ^50^. Subsequent signal transduction and changes in gene expression differ substantially between these two pathways and are linked to assembly of different downstream signaling complexes (“Myddosome” versus “Triffosome”) that result in different immune responses ^10, 51, 52^. This includes activation of mitogen-activated protein (MAP) kinases and nuclear factor kappa B (NF-κB) following assembly of Myddosomes and induction of type 1 interferon and interferon-inducible genes following assembly of Triffosomes. This has triggered great interest in how MyD88- versus TRIF-dependent signaling by LPS-activated MD-2/TLR4 may be differentially regulated.

CD14 can play an important role in TRIF-dependent signaling by coupling LPS recognition by CD14 to endocytosis of the surrounding membrane region ^53^. Cell surface MD-2/TLR4 (either wild-type or TLR4 mutated to preclude TLR4-dependent signal transduction) but not TLR4 alone can be internalized in an LPS- and CD14-dependent manner, consistent with a key role of MD-2 but not MD-2/TLR4 activation in LPS/CD14-driven endocytosis of MD-2/TLR4 ^54^. This MD-2 dependence may be explained by LPS-induced CD14–MD-2 interactions that facilitate LPS transfer from CD14 to MD-2/TLR4 and bring MD-2/TLR4 into close proximity to mCD14. The added requirement of ternary LPS.MD-2.TLR4 complexes that readily dimerize ^54, 55^ is consistent with the likely enrichment of these complexes along with mCD14 in lipid rafts that slow diffusion of the ternary complexes away from mCD14 and associated endocytic machinery ^56^. Given that the dimerized ternary complexes are the proximal intermediates to both signaling pathways, it is not surprising that a myriad of mechanisms confer selective weighting to MyD88- or TRIF-dependent signaling in a cell type- and circumstance-dependent fashion ^12^. Inclusion of LPS within more complex particles—for example, OM vesicles (OMVs) and intact GNB—might also selectively promote TRIF-dependent (or different) TLR4 signaling ^57– 59^, possibly by engaging additional host cell endocytic and phagocytic machinery as well as retarding the extraction and delivery of LPS monomers so that ingested LPS-rich particles may be co-localized with intracellular ligand-free MD-2/TLR4. Of note, the apparent requirement for “aggregated” mCD14 in CD14-dependent endocytosis seems compatible with a mechanism that can be triggered upon interaction of mCD14 with an LPS-rich particle. Initial binding of extracellular LBP to these particles should provide binding sites for multiple molecules of mCD14 that, according to the findings of Ryu *et al*. ^9^, can be replenished as monomeric LPS.mCD14 complexes are released and engage MD-2/TLR4. Given the progressive nature of this process, it seems likely that endocytic cargo will include partially extracted remnants of the particles that can release LPS monomers within these intracellular compartments and engage—if present—stores of MD-2/TLR4. The linkage of CD14-triggered endocytosis to the physical (particle) state of LPS is appealing in that it would functionally link the uptake of antigen-rich particles (for example, OMVs) by antigen-presenting cells (APCs) to activation of TRIF-dependent signaling pathways instrumental in APC maturation ^60, 61^. The favorable adjuvant properties of liposomes or OMVs containing relatively weak MD-2/TLR4 agonists (for example, monophosphoryl lipid A or penta-acyl LPS ^62, 63^) may reflect the effects of (1) inefficient ternary complex dimerization ^64^, (2) increased endocytic uptake, and (3) selective autocrine and paracrine effects of TRIF-dependent interferon-β pathway ^65^.

## How is LPS presented to MD-2/TLR4 during infection?

Despite years of intense study of LPS–MD-2/TLR4 interactions, how LPS is presented to MD-2/TLR4 during infection (that is, as an integral OM component) has not been adequately studied. Comparative studies with purified meningococcal OMV versus LPS-agg showed the same requirement for LBP/CD14 to extract and deliver LPS monomers to MD-2/TLR4 ^18^. Differences in potency of equivalent amounts of LPS presented either as LPS-agg or in OMV were entirely attributable to differences in efficiency of formation of monomeric LPS.CD14 complexes (LPS-agg > OMV) (that is, delivery of LPS monomer to MD-2/TLR4). Thus, variables in the interfacial properties of LPS-rich surfaces could markedly alter LPS potency as an MD-2/TLR4 agonist (or antagonist). This could include variables in LPS structure (for example, acylation and charge) or other OM components affecting LPS packing and surface charge or both. Whether or not OMVs are important intermediates in MD-2/TLR4 activation by LPS during infection requires further study.

## Caspases surveil the cytosol

Inflammasomes are multi-protein complexes that mediate inflammatory cell death (pyroptosis) in response to invasion by microbes or to the presence of altered endogenous molecules or intracellular structures ^66^. For canonical inflammasomes, ligand-binding proteins assemble with adapter proteins and culminate in the multimerization and catalytic auto-activation of caspase-1 ^67^. By contrast, the non-canonical inflammasome, which mediates the response to cytosolic LPS, activates distinct inflammatory caspases (-11 in mice and -4 and -5 in humans) ^2, 4, 6, 68– 74^, resulting in the induction of pyroptosis following cleavage of gasdermin D and maturation and secretion of interleukin 1 beta (IL-1β) and IL-18 following activation of NLRP3-dependent canonical inflammasomes. Given what was known about canonical inflammasomes, it was a surprise to find that caspase-4/-5/-11 could be activated directly by purified LPS in the absence of other host proteins, suggesting an activation mechanism in which the caspase is both sensor and effector ^5^. The fundamental challenge faced by cytosolic caspases is in part the same as for the MD-2/TLR4 system: how does the host recognize specific structural features of a lipid that is normally buried in the GNB OM? Because of the cytosolic location of the LPS-sensing caspases, additional questions include (1) how is caspase-activating LPS delivered to cytosolic caspases and (2) how are LPS structure recognition and caspase activation achieved? Although the non-canonical inflammasome seems to share with MD-2/TLR4 a preference for hexa-acyl phosphorylated lipid A/LPS ^4– 6, 75^, the data that we review below suggest that the mechanisms for LPS-induced non-canonical inflammasome activation are different from those of the MD-2/TLR4 system.

## What form of LPS reaches the cytosol and activates caspase-4/-5/-11?

**Figure 2.**
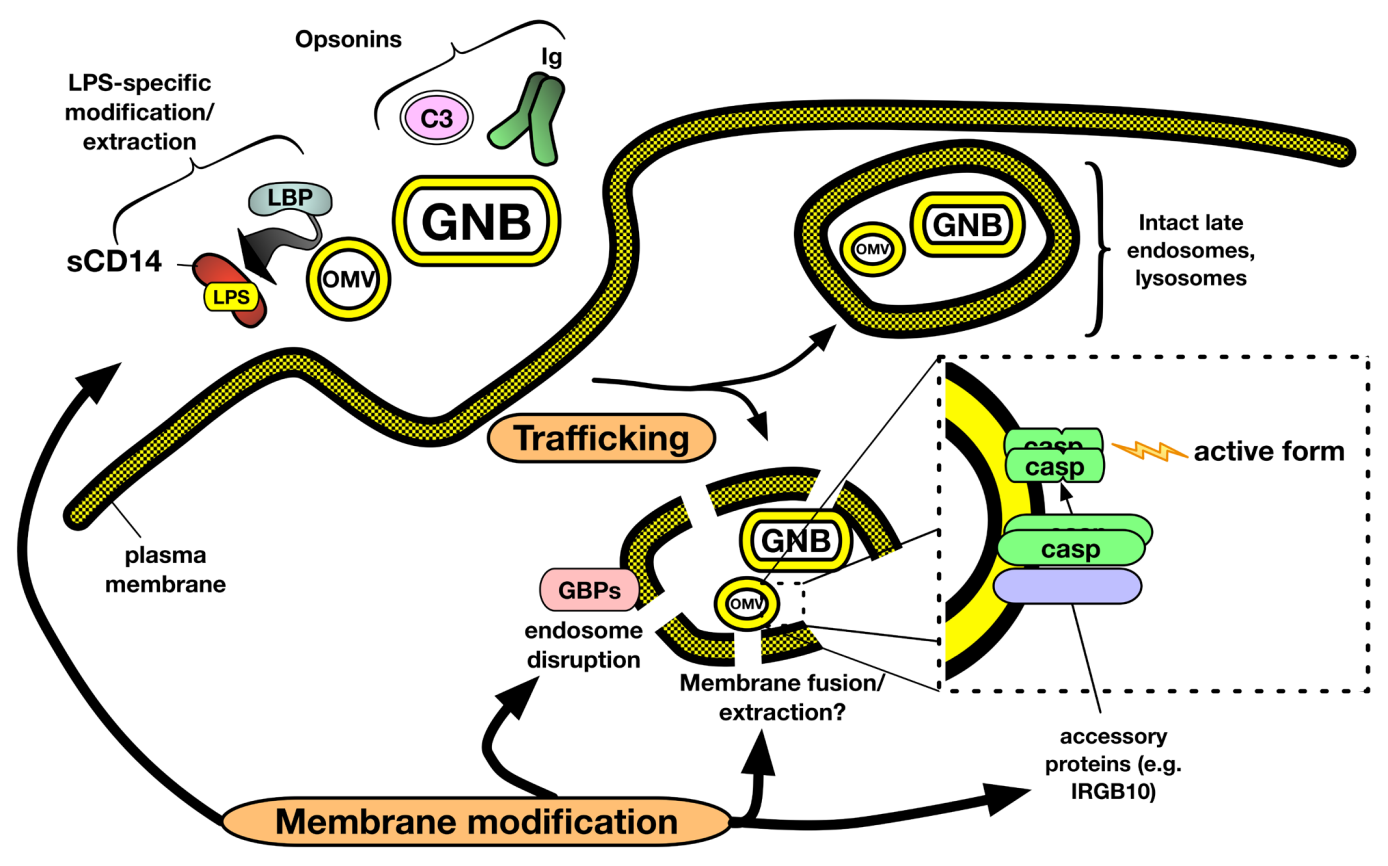
Schematic of potential determinants of delivery of activating LPS to non-canonical inflammasome caspases. We hypothesize that both trafficking and membrane modification events regulate delivery of LPS to cytosolic caspases. In addition to responses mediated by the MD-2/TLR4 system ( Figure 1), extracellular OMV and GNB encounter extracellular and cell surface host proteins that alter the microbial surface through both lipid extraction (for example, LBP and CD14) and opsonization (for example, complement and antibodies). These interactions, together with intrinsic distinguishing structural features of OMV and GNB, result in trafficking of LPS-containing membranes to compartments in which membrane disruption facilitated by host proteins (for example, guanylate-binding proteins or GBPs) leads to exposure of modified LPS-rich outer membrane to the cytoplasm. Alternatively, membrane fusion between OMV/GNB and host membranes, followed by translocation of LPS molecules to the cytoplasmic face of the host vacuolar membrane, may generate a caspase-activating surface. Oligomerization of surface-bound caspases occurs at one or more of these LPS-rich membrane interfaces, resulting in auto-catalytic activation and downstream cellular alterations. GNB, Gram-negative bacteria; LBP, lipopolysaccharide-binding protein; LPS, lipopolysaccharide; OMV, outer membrane vesicle.

Prevailing models of LPS-induced caspase activation posit formation of monomeric LPS.caspase complexes and subsequent multimerization and activation ^2, 76^. However, as yet, there is no supporting evidence for this model. The amphipathic properties of LPS molecules necessitate the association of LPS molecules with other amphipathic molecules, as occurs within the GNB OM and aggregates of purified LPS, or with hydrophobic binding pockets of specific LPS monomer-binding proteins (for example, CD14, albumin, and MD-2; see above). Characterization of complexes formed between purified meningococcal LPS aggregates and a catalytically dead mutant (C258A) of caspase-4 to model initial interaction of pro-caspase with LPS revealed very large supra-molecular aggregates (Mr >> 300,000) with about 1 mol caspase bound per 10 mol LPS ^77^. Interaction of caspase with LPS resulted in no apparent extraction or recovery of 1:1 LPS.caspase complexes in either monomeric or multimeric form. We thus favor a model of LPS engagement and auto-catalytic activation of LPS-sensing pro-caspases that is more akin to that of Factor C in the horseshoe crab *Limulus* rather than MD-2/TLR4, in which LPS-rich surfaces rather than LPS.protein monomers are the trigger of protein multimerization and activation ^78^ ( Figure 2). The possibility that an LPS-rich surface can provide an organizing scaffold that aligns bound caspase molecules for subsequent auto-activation could obviate the need for distinct scaffold proteins.

Existing data suggest at least two different routes by which bacteria-derived LPS can gain access to and activate cytosolic caspases: (1) access of bacteria to the cytosol of infected cells ^69, 79– 82^ and (2) endocytosis of OMV shed by extracellular GNB ^83, 84^. As yet, direct interaction of LPS-sensing caspases to intact (replicating) GNB has not been tested. Binding of pro-caspase-4 to OMV purified from growing GNB (meningococci) has been demonstrated ^77^ but how general this phenomenon is and whether it leads directly to caspase activation require further investigation, especially given the likely heterogeneity of OMV derived from different GNB and in different environmental settings ^85– 87^. Recent studies have demonstrated an important role for interferon-induced guanylate-binding proteins (GBPs) in caspase-4/-11-dependent pyroptosis and NLRP3 inflammasome activation triggered by both infecting GNB and endocytosed OMV ^75, 81, 82, 88, 89^. Microscopy of ingested bacteria suggests that GBPs serve to disrupt microbe-containing vacuoles, thus permitting exposure of LPS to the cytosol ^81, 82, 89^. Other cytosolic proteins may assist in modification of the GNB OM for LPS recognition by the non-canonical inflammasome. Recent data show that the interferon-inducible protein IRGB10 can target and disrupt cytosolic GNB in concert with GBPs and that knockout of IRGB10 attenuates caspase-11-dependent pyroptosis in response to *Escherichia coli* infection ^81^. These data suggest that presentation of LPS in intact GNB or shed OMV requires modification of the bacterial OM for optimal activation of the non-canonical inflammasome.

## “Pattern recognition” by caspase-4/-5/-11

As described above, distinguishing structural properties of LPS monomers (for example, hexa- versus tetra-acylated LPS) can determine the agonist versus antagonist properties of LPS toward MD-2/TLR4. Such differences in LPS acylation can also alter the supra-molecular organization of LPS-rich surfaces by affecting the geometry of the lipid A of individual LPS molecules. Specifically, LPS-rich surfaces composed of activating hexa-acylated LPS are more likely to form inverted cubic or hexagonal supra-molecular structures because of the greater area occupied by the six acyl chains versus the polar head group of lipid A. Conversely, tetra-acylated LPS are more cylindrical in shape and form multilamellar supra-molecular structures ^90– 92^. These differences in supra-molecular structure do not appear to affect the efficiency of LBP/CD14-mediated extraction and delivery of hexa- versus tetra-acylated LPS monomers and hence do not contribute to the differences between hexa- and tetra-acyl LPS in MD-2/TLR4 activation ^49^. However, if LPS-rich surfaces rather than LPS monomers are the activating form of LPS for cytosolic caspases, it is conceivable that variables in the packing of LPS at membrane interfaces affect the spacing of bound caspase and hence the probability of caspase activation.

Purified LPS is a much less potent inducer of caspase-4/-11-dependent pyroptosis than OMV, even when LPS content is normalized ^83, 84^. Thus, although purified LPS can activate purified non-canonical inflammasome caspases, LPS in purified form apparently lacks (as-yet-undefined) structural or supra-molecular features necessary for productive cellular uptake, intracellular delivery, or interaction with cytosolic caspase or a combination of these. Fusion of OMV with host membranes has previously been invoked to explain the delivery of virulence factors to host membranes or to the cytosol ^93– 95^. Translocation of LPS from bacterial membranes into or through host endosomal or phagosomal membranes may require not only specific host factors (for example, dynamin-like GBPs ^96, 97^) but also specific interfacial properties of the bacterial surface containing LPS. Future *in vitro* studies of caspase activation with various purified LPS, OMV, and subcellular fractions of host cells that had taken up OMV should help better define the LPS structural and supra-molecular requirements for LPS-induced caspase activation.

## Summary

The discovery of LPS-sensing caspases was initially aided by the use of TLR4-null host test systems to unambiguously demonstrate the existence of an additional pro-inflammatory LPS recognition/response system in mammalian hosts ^4, 6^. Although TLR4-independent function of LPS-sensing caspases has been reported by many, the ability of extracellular OMV to engage and activate both cell surface and endosomal MD-2/TLR4 ( Figure 1) and cytosolic caspase ( Figure 2) suggests that the action and regulation of these distinct LPS recognition/response systems can be integrated. For example, the capacity of mCD14 to couple endocytosis of LPS-rich particles (for example, OMVs) with activation of endosomal MD-2/TLR4 could contribute to both biochemical changes in the host cell cytosol (via TRIF-dependent signal transduction) and structural changes of the GNB OM (secondary to extraction of OM LPS and phospholipid monomers ^18^) that are instrumental to caspase activation. As has been the case for studies of LPS-mediated MD-2/TLR4 activation, better understanding of the mechanisms mediating caspase activation by LPS will likely facilitate future discoveries of host and bacterial properties that can regulate caspase activation by LPS.
